# Impact of simultaneous exposure to RF and gradient electromagnetic fields on implant MR safety labeling

**DOI:** 10.1002/mrm.70059

**Published:** 2025-08-29

**Authors:** Umberto Zanovello, Alessandro Arduino, Carina Fuss, Tolga Goren, Luca Zilberti, Oriano Bottauscio

**Affiliations:** ^1^ Istituto Nazionale di Ricerca Metrologica Torino Italy; ^2^ IT'IS Foundation Zurich Switzerland

**Keywords:** gradient heating, implant safety, MRI safety, radiofrequency heating

## Abstract

**Purpose:**

To investigate whether heating contributions produced by radiofrequency (RF) and gradient fields superpose sufficiently at the worst‐case locations to justify their simultaneous consideration in magnetic resonance imaging (MRI) implant safety labeling.

**Theory and Methods:**

Six implant models were positioned in an ASTM phantom and realistically implanted in two anatomical human models, and exposed to gradient and RF fields at 64 MHz and 128 MHz. The simulations with the anatomical body models considered different axial exposure landmarks inside the RF and gradient body coils. The exposures were scaled to represent two sets of scenarios: either limited by the implant's MR conditional labeling to a fixed peak temperature rise, or representing an EPI or TrueFISP examination with clinically relevant parameters, where the implant label is not limiting.

**Results:**

The temperature enhancement due to the combined RF and gradient sources, evaluated with respect to the maximum values obtained separately, depends on the implant, pulse sequence, and exposure landmark. A maximum relative enhancement of about 65% was found in the ASTM phantom, and maximum absolute enhancements above 0.3 K were found in anatomical models with realistic pulse sequences.

**Conclusion:**

There are clinically relevant MR examination scenarios where the maximum heating contributions produced by RF and gradient fields combine, enhancing the local peak temperature increase beyond that obtained from either assessment alone. The results prove to be useful for defining safety margins on the maximum allowable temperature increase, avoiding the requirement of a combined gradient coil and RF test.

## 1 INTRODUCTION

The heating of implants during a magnetic resonance (MR) examination due to the radiofrequency (RF) field and the gradient field are potentially additive,^1^, ^2^ yet the two phenomena are typically assessed independently^3^; their superposition is rarely considered in safety assessments, as it is not directly required in any standard or regulatory guidance. There are several reasons for this^1^, ^4^:

1. RF fields deposit energy directly in the biological tissues surrounding the implant, whereas gradient‐induced fields deposit energy in the metallic implant. The spatial distributions of the resulting temperature increases, as well as the hotspot locations around a given implant, are significantly different;
2. Gradient coil (GC) heating is usually stronger when the implant is relatively far from the isocenter, whereas RF heating is often stronger when the implant is close to the isocenter or to the end‐ring of the RF coil;
3. MR sequences, which are the worst‐case for GC heating, are also not typically the worst‐case for RF heating, and vice versa;
4. The test equipment of benchtop heating assessment, as well as the selected MR protocols for heating assessments in clinical scanners and the standards specifying the test protocols, are different and independently developed, encouraging their independent treatment. These methods are necessarily summarized down to heating rates, worst‐case temperatures, or thermal doses of local hotspots, for implants under unrelated test exposure scenarios. Therefore, no information about the 3D spatial/temporal distributions of heat is typically available;
5. The MR sequence parameters which can limit RF heating (SAR, $B_{1}^{+}$ rms), GC heating (dB/dt rms), or both (scan duration), are independently specified, thus RF‐GC heating superposition can be mitigated by adjusting any of the three parameters, creating difficulties for developing guidance.

These reasons can be subdivided into risk‐based considerations (items 1–3) and practical considerations (4–5). The increasing use of high‐fidelity numerical modeling of patient exposures using human body models and implant surrogates, in lieu of or in supplement to phantom‐based physical testing, is relevant to several of these points. The most significant practical consideration, item 4, is surmounted by access to the full 3D spatial distribution of local hotspots granted by numerical methods, increasing the feasibility of efficiently assessing combined RF‐GC heating. Numerical methods now allow potential safety risks to be reliably identified ahead of time through multi‐physics *in vivo* simulations, rather than waiting for a clinical report of an injury directly attributed to the phenomenon in question.

Under these circumstances, the question is whether the (now feasible) effort of considering combined RF‐GC heating is justified by real potential risks, that is, considering points 1–3, do realistic MR examinations produce simultaneous RF and GC heating which superposes sufficiently at the worst‐case locations to justify their consideration in implant safety labeling? The objective of this study is to definitively answer this long‐discussed question.

## 2 METHODS

The analysis develops in two directions. First, phantom simulations are performed complying with the requirements of the ASTM F2182^5^ and ISO 10974^6^ standards for testing of medical device heating exposed to RF and GC, respectively. Despite the ISO 10974 is a standard nominally conceived for testing of active implants, the same GC testing method is used here independently for passive and active implants. This first analysis is applied to all the considered implants with the exception of the active one (see section 2.1). For RF, the implants are positioned inside the ASTM phantom (see Supporting Information) in a region of homogeneous electric field generated by the selected RF coil, and the temperature increase is computed. GC simulations compute the temperature increase distribution by exposing the implants to a homogeneous and harmonic magnetic field, oriented along the direction leading to the highest heating.^3^ Both RF and GC simulations are independently scaled to an (arbitrary) peak local temperature limit, and then the two distributions are superposed. This scaling approach represents the maximum exposure permitted by MR conditional labeling derived from separate RF and GC heating assessments.

The second part of the study shifts the analysis from the testing framework toward a clinical context. Realistic body models are positioned at different axial landmarks within the RF and GC coils and exposed to the electromagnetic field generated by realistic MR pulse sequences. The human body exposure results are scaled in two ways. In the first scaling approach, RF and GC temperature increases are scaled to the same maximum value across the landmarks. As in the phantom, this represents exposure limited by MR conditional labeling. In the second scaling approach, EPI and TrueFISP pulse sequences are applied at clinically realistic parameters—that is, a typical examination within MR system limits and not considering any MR conditional label—and the resulting RF and GC temperature distributions are calculated and superposed.

The sections below describe the details of the analyses.

### 2.1 Implant models

The study analyzes five passive and one active implant selected according to their prevalence and likelihood of resulting in concerning heating due to RF or GC exposure.^2^, ^7^, ^8^, ^9^ Table 1 collects the implants considered in the study together with the manufacturer, model, and materials. Table S1 in the Supporting Information reports the electric and thermal properties of each material composing the implants. All the implant models accurately reproduce the geometries of the manufactured and commercialized counterparts. With the exception of the implantable pulse generator (IPG) module of the active implant, all the implants are intended for clinical use, and the manufacturer itself provided the virtual models. In order to account for an active implant without focusing on a specific model, the analysis included the generic SAIMD‐U testing device.^10^ The SAIMD‐U is a verification and validation device for evaluations according to ISO 10974, whose characteristics are described in the Annex U of the current ISO draft edition of the standard.^6^

**TABLE 1:** Implant models considered for the GC and RF heating analysis.

Implant	Manufacturer	Model	Material
Hip	Adler Ortho® SpA	Stem & Ball: Apta‐Fix	Stem & Ball & Acetabular Cup: CrCoMo
		Acetabular Cup & Liner: Fixa Ti‐Por	Liner: UHMWPE
Knee	Adler Ortho® SpA	Genus MB	Femoral & Tibial Plate: CrCoMo
			Liner: UHMWPE
Shoulder	LimaCorporate SpA	SMR Anatomic	CrCoMo
Cranial Plate	Medartis AG	Mesh, M2‐7093S	Ti‐6Al‐4V
Ankle Plate	Medartis AG	TriLock Distal Tibia, A‐4954.25S Left	Ti‐6Al‐4V
Active Device	Zurich Med Tech	SAIMD‐U	IPG Can: Stainless Steel
			IPG Hollow Parts: Air

### 2.2 Anatomical body models and implant positioning

The selected body models are the result of a survey aimed at investigating the best match between the anatomy and the available implant models. The different anatomical details require paying particular attention to the size of each implant component in order to fit the specific human model. The Glenn (adult male, 84 years old, 183 cm height, 20 kg m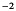 BMI) and YoonSun (adult female, 26 years old, 152 cm height, 23.6 kg m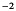 BMI) human models of the Virtual Population^11^ best fitted the available implants and were therefore chosen. All simulations involved Glenn except those including the shoulder implant; since, for compatibility reasons, it was not possible to implant the shoulder prosthesis inside Glenn, the analysis focused on YoonSun when such an implant was involved. As a consequence of the impact that the periprosthetic tissues have on the RF heating,^12^ the implants were not simply overlapped to the original body model; instead, a more accurate procedure was followed by reshaping the bones in order to adequately accommodate the relevant implant. Wherever voids originated from this procedure, these were assigned the electric and thermal properties of connective tissues.^13^ The ankle plate also required the addition of a layer of skin to avoid unrealistic contact of the implant with the surrounding air/background. The analysis considered all left‐side implants for Glenn and a right‐side shoulder prosthesis for YoonSun.

### 2.3 Gradient and radiofrequency coils

A full body GC set, representing tubular magnetic resonance imaging (MRI) scanners GCs (Solaris‐R by Nanjing Cichen), generated the time‐varying gradient magnetic field. The GC set comprised three axial coils, which constituted the X, Y, and Z GCs used in clinical scanners. The overall length was 1.5 m, and the internal diameter was 67 cm. RF simulations involved a 16‐rung birdcage body coil with a 60 cm diameter and 70 cm length. This specific coil was selected among those available in the BCLib (ZMT)^14^ database according to its geometrical compatibility with the available GC set. The birdcage was selectively tuned at 64 and 128 MHz for operation at 1.5 T and 3 T, respectively.

### 2.4 Axial exposure landmarks

**FIGURE 1:**
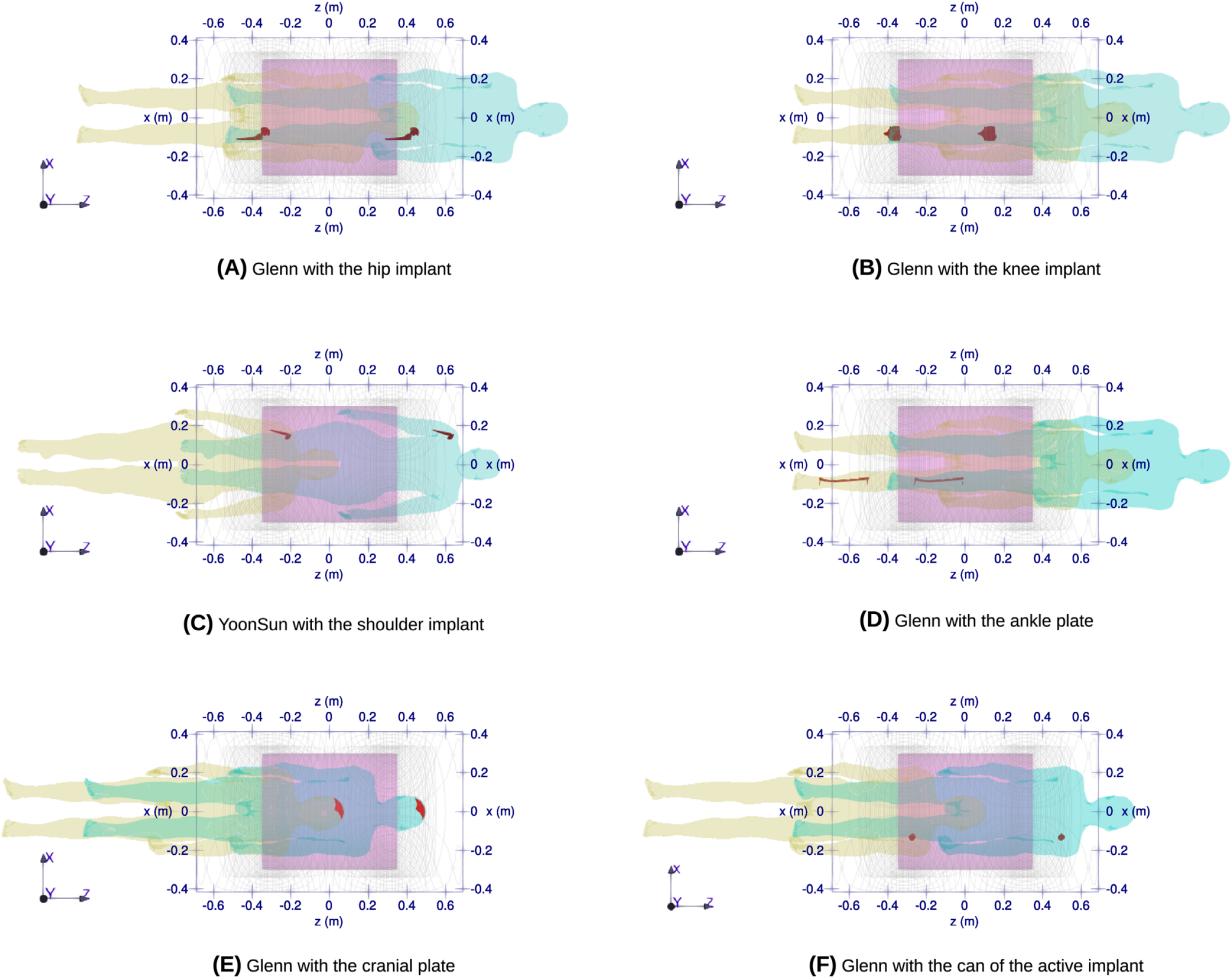
Range of exposure landmarks for different body/implant configurations. The figure depicts the extreme body positions within which the body models were moved in 7 cm steps. The pink transparent box and the black quoted outline represent the RF and GC, respectively (A) Glenn with the hip implant (B) Glenn with the knee implant (C) YoonSun with the shoulder implant (D) Glenn with the ankle plate (E) Glenn with the cranial plate (F) Glenn with the can of the active implant.

Simulations with anatomical body models accounted for a range of exposure landmarks selected for each body/implant combination. The range was defined by moving the body models in 7 cm steps along the axial direction of the coils until either the implant ended up outside the RF coil or the configuration resulted in an unrealistic imaging setting. When the number of simulated landmarks for an implant was less than seven, the analysis extended the range even outside the RF coil. Figure 1 depicts the landmarks range for all model/implant configurations.

### 2.5 MR pulse sequences

The analysis considered two MR pulse sequences, EPI and TrueFISP. The selected sequences are standard sequences included in most MRI protocols, and lead to a comparable exposure to RF and GC, in terms of temperature increase, for a range of landmarks. Table 2 reports the main settings of the sequences. The EPI was further divided into EPI‐X, EPI‐Y, and EPI‐Z, where X, Y, and Z identify the direction of the frequency encoding gradient. The same gradient waveforms were applied to both 1.5 T and 3 T scenarios, differing only in RF carrier frequency.

**TABLE 2:** Main parameters of the sequences considered for the analysis. Maximum Whole‐Body (WB) averaged specific absorption rate (SAR) refers to the maximum, including all implants and exposure landmarks for the specific sequence.

	EPI	TrueFISP
Flip angle	90∘	45∘
RF pulse duration (ms)	1.6	1
Time‐BW product	4	4
TE (ms)	21	3.2
TR (ms)	43	6.4
Readout BW (kHz)	150	126.3
Matrix dimension	64 × 64	256 × 256
FOV (mm 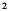 )	182 × 182	120 × 180
$B_{1,\text{rms}}^{+}$ (μT)	1.25	2.05
Maximum WB SAR @ 1.5 T, 3 T (W kg 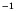 )	0.24, 0.71	0.65, 1.91
Maximum Gradient Amplitude (mT m 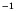 )	22.0	25.6
Maximum Gradient Slew Rate (T m 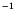 s 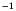 )	167	200
dB/dt rms @ r = 20 cm (T s 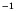 )	40.6, 40.6, 34.0	33.0

*Note*: The three dB/dt rms values reported for the EPI sequence refer to the X, Y, and Z readout directions, respectively.

### 2.6 Gradient simulations

Gradient simulations were performed in Sim4Life^15^ and with a home‐made validated code,^16^ when simulations involved the ASTM phantom and anatomical body models, respectively. A 2 mm voxel resolution was seen to be sufficient to achieve numerically stable results, and the simulation domain was restricted to the region of the metallic implants since the power deposited by the GCs in the phantom and body tissues is negligible.^4^

In simulations involving the ASTM phantom, the implants were exposed to a harmonic, spatially homogeneous, magnetic field directed along the direction that maximizes the induced heating. An algorithm implemented in Sim4Life determined the worst exposure direction,^3^ and simulations were performed at 1750 Hz as suggested by the ISO 10974 Standard.^6^ These simulations exposed the implants to an arbitrary power, as the temperature increases were scaled to a reference peak value at a later stage (see section 2.8).

Simulations involving MR pulse sequences and realistic body models followed the structure discussed in Arduino et al.^16^ and complied with the implementation described by Bottauscio et al.^17^ As a consequence of the frequency content of the gradient waveform harmonic spectrum in the considered MR sequences, electromagnetic (EM) simulations modeled the skin effect in order to achieve more reliable results.

### 2.7 Radiofrequency simulations

RF simulations were performed using the verified Electromagnetic Finite‐Difference Time‐Domain (EM‐FDTD) solver of Sim4Life V7.2.^15^ All the simulations were performed at 64 MHz and 128 MHz with the coil at circular polarization.

First, the implants were positioned within the ASTM phantom, in a region of homogeneous electric field generated by the birdcage coil. The RF coil exposed the implants to an arbitrary power, as the temperature increases were scaled to a maximum reference value at a later stage (see section 2.8).

Simulations involving MR pulse sequences and realistic body models followed a two‐step Huygens' box approach. First, 2‐port simulations, representing the I and Q channel excitations of the unloaded birdcage coil, were performed, and the resulting electromagnetic fields were recorded with a suitable Huygens' box. Next, these recorded fields were used to generate equivalent exposure sources for the simulations of the implanted human body models Glenn and YoonSun, where the implant material was simulated as a Perfect Electric Conductor (PEC). For each implant, a bounding box enlarged by 10 mm on each side was defined, and the included domain was discretized with 1 mm isotropic voxels, with the exception of the cranial plate, which required 0.5 mm voxels in order to be reliably simulated. The remaining part of the human body was resolved with a resolution of 2 mm. The IQ channels were combined to achieve circular polarization and scaled to a $B_{1}^{+}$ of 1 μT in the slice at the isocenter of the coil.

The SAIMD‐U, as defined in Annex U of the latest draft of the ISO 10974 Standard,^6^ represents a generic leaded device. For incident RF fields, the SAR hotspot is usually found at the lead tip. The lead was thus simulated in a homogeneous body‐average tissue simulating medium with a linearly polarized plane wave source 150 mm from the tip with the E‐field tangential to the lead, as shown in Annex I of.^6^ The distribution around the lead tip was then placed at an appropriate position in the body of a human model and scaled to the appropriate power values (integrated over the −30 dB contour around the implant tip). The individual power values for each position were preliminarily determined by performing a Tier 3 analysis with the IMAnalytics software,^18^ using the scaled SAIMD‐U transfer function from Annex U of the ISO 10974 Standard, a generic pacemaker routing and the quadrature $B_{1}^{+}$ distributions, simulated in the human model without any implant, scaled to an average magnitude of 1 μT in the slice at the isocenter.

### 2.8 Thermal simulations

The power density distributions resulting from GC ($P_{\text{GC}}$) and RF ($P_{\text{RF}}$) simulations were extracted on a regular 2 mm grid. As a consequence of the electromagnetic model, $P_{\text{GC}}$ and $P_{\text{RF}}$ were exactly zero in the tissues or in the metallic parts of the implant, respectively. During GC exposure, the tissues heat up indirectly, due to thermal diffusion from the metallic components of the implant, whereas during exposure to RF, the heat diffuses from the tissues to the implant, where the direct thermal effect of RF fields is negligible.^4^, ^19^ Pennes' bioheat equation was expressed in terms of temperature elevation with respect to the temperature at rest and solved, by means of a home‐made code,^16^, ^20^ accounting for $P_{\text{GC}}$ or $P_{\text{RF}}$ as sources. In the case of the anatomical body models, the bioheat equation also modeled the blood perfusion in the tissues, according to the parameters reported in the database of the IT'IS Foundation.^13^ This led to two temperature increase distributions, one due to GC alone and another to RF alone. Finally, the temperature increase due to the simultaneous presence of gradient and RF effects was obtained as the summation of the previous GC and RF temperature distributions. Thanks to the linearity of the Pennes' equation, this corresponded to solving the problem using the sum of $P_{\text{GC}}$ and $P_{\text{RF}}$ as the forcing term.

The analysis with the ASTM phantom combined, point by point, the spatial distributions of the temperature increases produced by the GC and RF fields, scaled to the same peak value within the phantom.

Simulations involving the MR pulse sequences and realistic body models scaled $P_{\text{GC}}$ according to the running sequence waveform and $P_{\text{RF}}$ according to the required flip angle, RF pulse duration, and modulation envelope (i.e., a *sinc* function with 0.5 apodization coefficient).^1^, ^2^ Both $P_{\text{GC}}$ and $P_{\text{RF}}$ were averaged over the repetition time (TR) of the sequence and used as a source of the thermal problem. The GC and RF temperature increases were combined in two ways: the first by summing GC and RF temperature increases scaled to the same maximum value across the landmarks, and the second by summing GC and RF temperature increases as obtained from the actual $P_{\text{GC}}$ and $P_{\text{RF}}$ generated by the relevant MR pulse sequence.

All temperature increases are provided as peak values across the computation domain, for a 30 min exposure to the different power sources.

## 3 RESULTS

Table 3 collects the relative enhancements of the peak temperature increases in the ASTM phantom due to the simultaneous exposure to GC and RF fields, with respect to the peak temperature increase obtained with GC or RF alone, scaled to the same reference value. Relative enhancements range from 17% to 67% at 1.5 T and from 24% to 51% at 3 T, depending on the implant.

**TABLE 3:** Relative enhancements of the maximum temperature increase due to the simultaneous application of GC and RF fields in an ASTM phantom, with respect to the temperature increase due to GC or RF alone.

Implant	Relative enhancement (%)	
	1.5 T	3 T
AnklePlate	17	28
CranialPlate	67	51
Hip	29	40
Knee	39	24
Shoulder	21	23

*Note*: RF and GC temperature increases were scaled to the same peak value before being combined.

The exposure conditions of the implants inside the ASTM phantom are quite different from those in a clinical setup. This reflects on the temperature increase enhancements obtained when the analysis involved MR pulse sequences and realistic body models, and the GC and RF temperature increases were scaled to the same maximum values across all landmarks before being combined. In this regard, Table 4 collects the relative enhancements of the peak temperature increases in the cases of realistic MR pulse sequences and anatomical human body models. The results show that the maximum relative enhancements never exceed 9% and 11% at 1.5 T and 3 T, respectively. Table S2 in the Supporting Information reports the landmarks where the maximum temperature increases are reached by RF, GC, and their combination. It is interesting to notice that, most of the time, the maximum temperature increase due to the simultaneous exposure to GC and RF occurs at the same landmark as in the case of exposure to the GC field alone.

**TABLE 4:** Relative enhancements of the maximum temperature increase due to the simultaneous application of GC and RF fields with the anatomical body models, with respect to the temperature increase due to GC or RF alone.

Implant	Sequence	Relative enhancement (%)	
		1.5 T	3 T
AnklePlate	EPI‐X	0.52	0.95
	EPI‐Y	0.12	1.35
	EPI‐Z	1.05	1.56
	FISP	0.52	0.95
CranialPlate	EPI‐X	1.53	4.36
	EPI‐Y	1.72	4.11
	EPI‐Z	0.85	2.85
	FISP	0.91	3.37
Hip	EPI‐X	2.53	2.47
	EPI‐Y	4.24	4.00
	EPI‐Z	3.06	3.11
	FISP	1.85	1.70
Knee	EPI‐X	5.70	10.19
	EPI‐Y	8.17	4.49
	EPI‐Z	4.20	4.97
	FISP	4.20	4.97
SAIMD‐U	EPI‐X	5.97	7.62
	EPI‐Y	5.97	7.62
	EPI‐Z	3.30	4.55
	FISP	5.96	7.61
Shoulder	EPI‐X	7.58	7.20
	EPI‐Y	8.49	9.57
	EPI‐Z	7.42	8.47
	FISP	6.92	6.82

*Note*: For each implant and MR pulse sequence, RF and GC temperature increases were scaled to the same maximum value across the landmarks.

**FIGURE 2:**
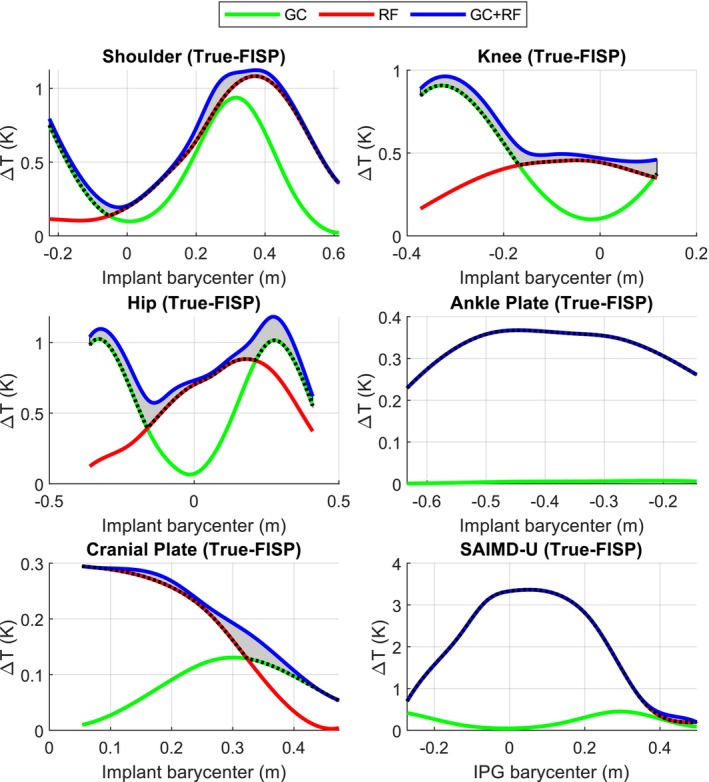
Peak temperature increase, in kelvin, on the six implants exposed to different MR pulse sequences as a function of multiple axial landmarks. Results refer to 1.5 T exposure to the RF EM field alone (red line), GC EM field alone (green line), and simultaneous application of RF and GC EM fields (blue line). The gray area highlights the enhancement of the peak temperature increase due to the simultaneous exposure to RF and GC EM fields with respect to the maximum between the peak temperature increase due to RF or GC alone.

**FIGURE 3:**
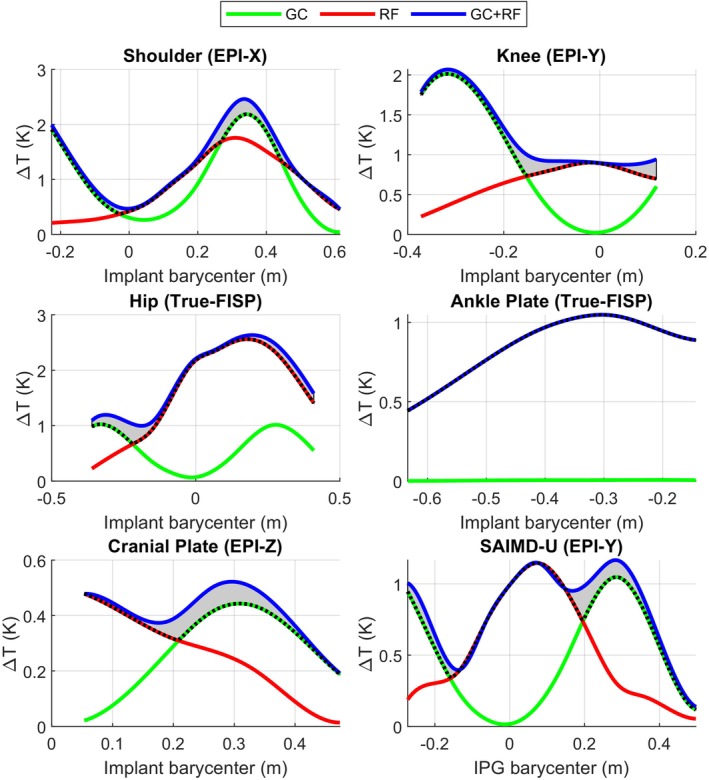
Peak temperature increase, in kelvin, on the six implants exposed to different MR pulse sequences as a function of multiple axial landmarks. Results refer to 3 T exposure to the RF EM field alone (red line), GC EM field alone (green line), and simultaneous application of RF and GC EM fields (blue line). The gray area highlights the enhancement of the peak temperature increase due to the simultaneous exposure to RF and GC EM fields with respect to the maximum between the peak temperature increase due to RF or GC alone.

Figures 2 and 3 show the peak temperature increase for 1.5 T and 3 T scenarios, respectively, resulting from the exposure to the specific MR pulse sequences whose parameters complied with those listed in Table 2. The figures report the temperature increases as a function of the axial position of the implant barycenter with respect to the scanner isocenter. The selection of the reported results was performed on a case‐by‐case basis, according to the combination of implant and sequence leading to the highest enhancements of the temperature increase due to the simultaneous effect of RF and GC EM fields with respect to RF and GC alone. This choice did not necessarily lead to showing the cases where the absolute maximum temperature was reached. Nevertheless, the maximum peak temperature increase also played a role in excluding those scenarios where the heating due to both RF and GC was less prominent. Figures analogous to Figures 2 and 3 are provided as Supporting Information for all combinations of implant and MR pulse sequences (see Supporting Information). The gray areas in the figures highlight the enhancement of the peak temperature increase when the RF and GC EM fields are applied simultaneously with respect to the maximum between the peak temperature increase due to the application of RF or GC EM field alone. Scenarios with the greatest distance between the blue and the dashed lines represent those where the addition of the two effects has the largest effect on the combined temperature increase. In this regard, Table 5 collects the maximum peak temperature increase enhancements for each exposure scenario. The table reports the enhancements as the peak temperature increase following the combined application of GC and RF, reported next to the maximum between the peak temperature increase due to RF or GC alone.

**TABLE 5:** Maximum peak temperature enhancements, among all the landmarks for each specific scenario. Each table entry reports the temperature increase due to RF or GC alone (the highest of the two as reported in the brackets) on the left of the temperature increase due to the combined effect of RF and GC.

	EPI‐X	EPI‐Y	EPI‐Z	TrueFISP
Shoulder	1.64 (GC)—1.71 [0.07]	0.59 (GC)—0.67 [0.08]	1.29 (GC)—1.35 [0.06]	**0.89 (RF)—1.03 [0.14]**
	**1.67 (RF)—2.00 [0.33]**	0.44 (GC)—0.53 [0.09]	1.28 (RF)—1.39 [0.11]	0.75 (GC)—0.97 [0.22]
Knee	0.38 (GC)—0.42 [0.04]	0.15 (RF)—0.22 [0.07]	0.41 (GC)—0.45 [0.04]	**0.43 (RF)—0.52 [0.09]**
	0.70 (RF)—0.82 [0.12]	**0.70 (RF)—0.94 [0.24]**	0.75 (GC)—0.87 [0.12]	1.08 (RF)—1.29 [0.21]
Hip	0.33 (GC)—0.42 [0.09]	0.79 (GC)—0.87 [0.08]	0.21 (RF)—0.30 [0.09]	**1.01 (GC)—1.18 [0.17]**
	1.01 (GC)—1.25 [0.24]	1.49 (GC)—1.73 [0.24]	1.23 (GC)—1.47 [0.24]	**0.68 (GC)—1.03 [0.35]**
AnklePlate	—	—	—	—
	—	—	—	**0.96 (RF)—0.97 [0.01]**
CranialPlate	0.14 (GC)—0.17 [0.03]	0.10 (GC)—0.14 [0.04]	**0.12 (GC)—0.15 [0.03]**	0.13 (GC)—0.17 [0.04]
	0.08 (RF)—0.11 [0.03]	0.27 (RF)—0.28 [0.01]	**0.41 (GC)—0.50 [0.09]**	0.73 (RF)—0.75 [0.02]
SAIMD‐U	0.56 (GC)—0.63 [0.07]	0.85 (GC)—0.93 [0.08]	0.50 (GC)—0.57 [0.07]	**0.24 (RF)—0.35 [0.11]**
	0.90 (GC)—1.07 [0.17]	**0.85 (GC)—1.03 [0.18]**	0.50 (GC)—0.62 [0.12]	—

*Note*: The temperature enhancement is added in square brackets near the temperature increase values. All temperature increases are reported in kelvin. For each implant, the first row refers to results at 1.5 T and the second row at 3 T. When the temperature increase enhancement resulted in being negligible for all the landmarks, a hyphen is reported in the corresponding table entry. Results shown in Figures 2 and 3 are reported in bold.

## 4 DISCUSSION

The Food & Drug Administration (FDA) suggests to include, along with the MR conditional labeling of a medical device, the maximum allowable gradient slew rate per axis, the maximum permitted WB averaged SAR, maximum permitted head averaged SAR, and the maximum $B_{1}^{+}$ rms admissible value.^21^ Constraining the maximum gradient slew rate reduces excessive GC heating, whereas limiting the maximum SAR and/or $B_{1}^{+}$ is useful to limit RF heating. Therefore, the previous quantities are restricted on the basis of testing results and a safety threshold in terms of maximum acceptable temperature increase. Independent of the actual value of the temperature increase threshold, when GC and RF testing are performed separately, applying independent limits is appropriate as long as the two heating effects do not combine significantly. When this holds true, if an MRI scan is performed, e.g., at the maximum $B_{1}^{+}$ value, the peak of the RF‐induced temperature increase may be close to the established threshold, if the clinical exposure is close to worst‐case. Of course, if at the same maximum $B_{1}^{+}$ value, the gradient also contributes significantly to the heating, the actual peak temperature increase may be higher than the safety threshold. As a consequence, if the interaction of RF and GC heating is not properly accounted for, maximum temperature increases could reach unexpected and unsafe values, exposing the patient to a potential hazard.

The analyzed implants were not labeled as MR conditional; therefore, the above information was not available, nor is there any generally accepted published value of maximum acceptable temperature increase. For these reasons, the study abstracted from these inputs, first by scaling the peak temperature increases obtained from GC and RF exposure to the same reference value, and then by considering an exposure to the EM field generated by MR sequences complying with normal operating mode and hardware watchdog limits.^22^

The first analysis with the ASTM phantom aimed at reproducing the standard tests used to assess heating of conductive devices due to RF^5^ and GC^6^ exposures. These tests are designed to limit RF and GC exposure levels on the basis of a defined maximum acceptable temperature increase. Independently of its actual value, it is reasonable to assume it is the same for RF and GC exposures, as it depends primarily on the sensitivity of the surrounding tissues. This is why the analysis combined GC and RF temperature increases after scaling them to the same peak reference value. Results indicate maximum temperature increase enhancements up to 67% and 51% at 1.5 T and 3 T, respectively.

The clinical applicability of phantom testing is limited in several respects. First, the RF testing exposure is quite different from that of a typical clinical scenario, where the electric field could be far from homogeneous. Second, the phantom's geometrical, electrical, and thermal properties differ from those of the human tissues. Finally, the ISO 10974 Standard^6^ for GC testing does not require determining the worst exposure direction on top of a previously calculated RF temperature increase. Therefore, there may be magnetic field directions leading to higher combined temperature increases, while causing lower temperature increases when the exposure is limited to GC alone. Thus, the phantom relative enhancements may under‐ or over‐estimate those reasonably obtainable in clinical conditions. For this reason, the analysis shifted to simulations of implants inside realistic body models, exposed to the EM field generated by actual MR pulse sequences. The MR sequences were selected for their common application in clinical protocols and comparable exposure to RF and GC sources in terms of peak temperature increase.

For all landmarks, the first analysis scaled the temperature increases resulting from the independent exposure to GC and RF to the same maximum reference value. This represents the scenario where the implants have been independently tested for GC and RF‐induced heating and maximum exposure parameters, such as dB/dt rms and $B_{1}^{+}$ rms, have been defined according to a maximum allowed temperature increase. Scaling the GC and RF temperature increases to the same maximum reference value across the landmarks emulates the scenario where the scan is limited by the implant's MR conditional performance limits, rather than being allowed to follow a typical clinical sequence. This led to a decrease of the maximum relative temperature increase enhancements to 8.5% and slightly above 10% at 1.5 T and 3 T, respectively, with respect to those obtained with phantom.

While this analysis is useful to investigate the temperature increase enhancements due to the combined GC and RF effects when both are at their safety limits, it also reflects a clinically unrealistic scenario where the GC and RF waveforms are designed to both reach the same maximum temperature increase. For this reason, the temperature increase due to GC and RF fields generated by realistic MR sequence parameters was also simulated. The sequence parameters were adopted from common scanning routines^2^ and complied with actual values of the gradient hardware watchdog limits.^23^ Whereas no information was available on the maximum safe $B_{1}^{+}$ rms for the considered unlabeled implants, the selected parameters complied with a scanner in normal operating mode, namely with WB SAR equal to or less than 2 W kg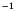. In particular, the maximum WB SAR amounted to 1.9 W kg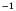 in the case of the hip and SAIMD‐U implants positioned at −0.29 m and 0.15 m from the isocenter, respectively. In both cases, the 1.9 W kg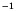 WB SAR value was obtained with the TrueFISP sequence at 3 T. It is worth specifying that the WB SAR does not represent a sufficient safety metric in presence of metallic implants, where the “antenna effect” should also be considered.^24^, ^25^ However, in the absence of any other information, the simulated value at least ensured that the sequences complied with safety limits in the absence of metallic implants. The gray areas in Figures 2 and 3 confirm that the temperature enhancement, due to the combined effect of GC and RF with respect to the maximum between GC or RF alone, depends on the specific scenario, that is, implant, sequence, and landmark. This comes as no surprise, since the shape of the object influences the GC and RF temperature increase distributions in a different way. In addition, the GC temperature increase distribution is also influenced by the direction of the gradient magnetic field, whose time evolution depends on both the landmark and the MR pulse sequence.

Furthermore, Figures 2 and 3 show that, for different implant positions, the maximum temperature increases due to GC and RF exposure alone are generally comparable. The ankle plate represents an exception since, as a result of its small cross‐sectional area orthogonal to the GC magnetic field Z‐direction, the heating due to GC is negligible. The highest temperature enhancements, as reported in square brackets in Table 5, show a maximum of 0.35 K in the case of the hip implant at 3 T with the TrueFISP sequence. This scenario also corresponds to the highest relative temperature enhancement, which amounts to 53%. Table 5, as well as Figures 2 and 3, show that the maximum enhancement can happen when either the GC or the RF peak temperature increase is dominant.

Table 5 reports the temperature increase in absolute terms, instead of relative ones as in the previous analysis. It is worth studying the absolute temperature increase because of the linearity of the considered bioheat equation, which implies that the computed temperature increase distribution due to the combined effect of GC and RF equals the sum of the temperature increase distributions due to CG and RF alone.^1^ When the EM field with the most severe thermal effect leads to a temperature increase sufficiently larger than the other, the spatial point where the temperature increase reaches its peak value will be the same for both the most severe and the combined cases. As a consequence, when the power related to the most severe effect increases, while the other remains constant, the absolute temperature enhancement in the combined case does not change. This condition occurs for all the scenarios collected in Table 5. In this perspective, the highest enhancements equal 0.33 K when RF is the most severe effect and 0.35 K when the most severe effect is GC. The first condition happens with the shoulder implant scanned with an EPI‐X sequence, and the second with the hip implant scanned with a TrueFISP sequence.

All the temperature increase results are reported after 30 min of exposure. This duration equals that suggested by the ISO 10974 Standard and corresponds to a conservative estimation of the typical scan duration.^6^ Preliminary investigations also showed that the temperature increase due to GC exposure is almost at steady state after 30 min.

The generality of the results presented in this paper is limited by the adoption of only one human model per implant. This limitation comes from the fact that the accurate implant positioning procedure adopted in this paper requires finding a proper match between the available implant and body models. The implant and body models available in the authors' database did not allow for a large number of degrees of freedom, especially considering that the body posture cannot be sensibly changed if the body model had to fit inside the available RF coil and GC set. Furthermore, the implant positioning procedure proved to be rather delicate, requiring, on several occasions, the assistance of a specialized orthopedic surgeon. For these reasons, the analysis mainly focused on the Glenn body model, representative of an elderly male and, as such, with an increased probability of bearing an implant. Another limitation of this work lies in the adoption of only one GC set and RF coil. Whereas multiple birdcage body coil models were available, only one GC model was available. In order to fit the RF body coil inside the 67 cm internal diameter GC, an RF coil with a 60 cm had to be selected.

## 5 CONCLUSION

The paper analyzes the peak temperature increase enhancements due to the simultaneous exposure of different medical devices to the RF and GC magnetic fields, with respect to the exposure to RF or GC alone. Simulations comprised an ASTM phantom and two realistic body models, a birdcage RF body coil operated both at 64 MHz and 128 MHz at circular polarization, a WB gradient coil system, and six implants accurately implanted inside the body models. Results show that relative enhancements of the peak temperature increase of more than 65% can be found in phantom measurements when GC and RF are combined. Maximum relative enhancements of the temperature increase of about 10% are obtained with the implants in the anatomical body models when scaled to the same MR conditional implant‐based performance limits. Finally, realistic EPI and TrueFISP sequence parameters led to maximum absolute temperature increase enhancements above 0.3 K and relative enhancements above 50%.

The outcomes prove to be useful for defining safety margins on the maximum allowable temperature increase, upon which the average $B_{1}^{+}$ rms and dB/dt rms are limited by the regulatory body in the MR conditional label of the medical devices. RF and GC heating test equipment, as well as test protocols for implant heating assessments, are different and independently developed. Building upon the provided results to account for the combined RF and GC effect in the definition of temperature safety margins would be a more practical solution than combining RF and GC in implant heating tests. Whereas this seems to be the most reasonable solution, the analysis may still be too limited in the number of simulated exposure scenarios. Reliable maximum temperature safety margins require extending the analysis to more implants, human body models, and MR pulse sequences.

## CONFLICT OF INTEREST STATEMENT

The authors declare no potential conflicts of interest.

## FUNDING INFORMATION

This study was supported by EMPIR Programme, Grant/AwardNumber: 21NRM05 STASIS; Metrology Partnership; European Union.

## Supporting information




**Data S1.** Supporting Information.

## References

[1] Arduino A , Zanovello U , Hand J , et al. Heating of hip joint implants in MRI: The combined effect of RF and switched‐gradient fields. Magn Reson Med. 2021;85:3447‐3462. 33483979 10.1002/mrm.28666 PMC7986841

[2] Clementi V , Zanovello U , Arduino A , et al. Classification scheme of heating risk during MRI scans on patients with orthopaedic prostheses. Diagnostics. 2022;12:1873. 36010224 10.3390/diagnostics12081873 PMC9406867

[3] Umberto Z , Carina F , Alessandro A , Oriano B . Efficient prediction of MRI gradient‐induced heating for guiding safety testing of conductive implants. Magn Reson Med. 2023;90:2011‐2018. 37382200 10.1002/mrm.29787

[4] Winter L , Seifert F , Zilberti L , Murbach M , Ittermann B . MRI‐related heating of implants and devices: A review. J Magn Reson Imaging. 2021;53:1646‐1665. 32458559 10.1002/jmri.27194

[5] Testing American Society, Materials . Standard test method for measurement of radio frequency induced heating on or near passive implants during magnetic resonance imaging. Standard F2182‐19e2: ASTM International West Conshohocken. ASTM International; 2020.

[6] International Organization for Standardization (ISO) . Assessment of the safety of magnetic resonance imaging for patients with an active implantable medical device. Standard ISO 10974, Draft: International Organization for Standardization (ISO). International Organization for Standardization (ISO); 2021.

[7] OECD . Commission European. Health at a Glance: Europe 2024. OECD Publishing; 2024.

[8] Pabinger C , Lothaller H , Geissler A . Utilization rates of knee‐arthroplasty in OECD countries. Osteoarthr Cartil. 2015;23:1664‐1673. 10.1016/j.joca.2015.05.008 26028142

[9] Knight SR , Aujla R , Biswas SP . Total hip arthroplasty ‐ over 100 years of operative history. Orthop Rev. 2011;3:e16. 10.4081/or.2011.e16 PMC3257425 22355482

[10] Zurich Med Tech . Universal Active Implantable Device for Systems Verification at 64 MHz and 128 MHz ; 2024. https://zmt.swiss/validation‐hw/saimd/saimd‐u/

[11] IT'IS Foundation . Virtual Population (ViP) Models ; 2024. https://itis.swiss/virtual‐population/virtual‐population/overview/

[12] Arduino A , Baruffaldi F , Bottauscio O , et al. Computational dosimetry in MRI in presence of hip, knee or shoulder implants: Do we need accurate surgery models? Phys Med Biol. 2022;67:245022. 10.1088/1361-6560/aca5e6 36541561

[13] Christian B , Philippe H , Fabienne DG , et al. IT'IS Database for Thermal and Electromagnetic Parameters of Biological Tissues, Version 4.2 ; 2024. https://itis.swiss/virtual‐population/tissue‐properties/database/dielectric‐properties/

[14] Cabot E , Zastrow E , Kuster N . Library of RF Exposure from Generic Birdcages for Comprehensive Implant‐Safety Evaluation ; 2025. https://itis.swiss/assets/Downloads/News‐Items/zastrowbclibismrm20163466.pdf

[15] Zurich Med Tech . Sim4Life . 2024. https://sim4life.swiss/

[16] Arduino A , Bottauscio O , Brühl R , Chiampi M , Zilberti L . In silico evaluation of the thermal stress induced by MRI switched gradient fields in patients with metallic hip implant. Phys Med Biol. 2019;64:245006. 31683262 10.1088/1361-6560/ab5428

[17] Bottauscio O , Chiampi M , Hand J , Zilberti L . A GPU computational code for eddy‐current problems in voxel‐based anatomy. IEEE Trans Magn. 2015;51:1‐4.26203196

[18] Zurich Med Tech . IMAnalytics ; 2024. https://sim4life.swiss/imanalytics

[19] Zilberti L , Zanovello U , Arduino A , Bottauscio O , Chiampi M . RF‐induced heating of metallic implants simulated as PEC: Is there something missing? Magn Reson Med. 2021;85:583‐586. 32936504 10.1002/mrm.28512

[20] Arduino A , Bottauscio O , Chiampi M , Zilberti L . Douglas‐Gunn method applied to dosimetric assessment in magnetic resonance imaging. IEEE Trans Magn. 2017;53:5000204.

[21] Food & Drug Administration . Testing and Labeling Medical Devices for Safety in the Magnetic Resonance (MR) Environment. Standard FDA‐2019‐D‐2837. Food & Drug Administration; 2023.

[22] International Electrotechnical Commission (IEC) . Medical Electrical Equipment ‐ Part 2–33: Particular Requirements for the Basic Safety and Essential Performance of Magnetic Resonance Equipment for Medical Diagnosis. Standard 60601–2‐33:2022. International Electrotechnical Commission (IEC); 2022.

[23] Mathias B . Gradient performance and gradient amplifier power. MAGNETOM Flash (69) 3/2017: Siemens Healthlineers. Siemens Healthineers; 2017.

[24] Shellock FG , Rosen MS , Webb A , et al. Managing patients with unlabeled passive implants on MR systems operating below 1.5 T. J Magn Reson Imaging. 2024;59:1514‐1522. 37767980 10.1002/jmri.29002

[25] Wooldridge J , Arduino A , Zilberti L , et al. Gradient coil and radiofrequency induced heating of orthopaedic implants in MRI: Influencing factors. Phys Med Biol. 2021;66:245024. 10.1088/1361-6560/ac3eab 34847533

