# Supplementary material for: Impact of simultaneous exposure to RF and gradient electromagnetic fields on implant MR safety labeling
Source: Magn Reson Med. 2025 Aug 29;95(1):601–12. doi: 10.1002/mrm.70059 (PMC12620153; doi:10.1002/mrm.70059)
Supplement: Supplementary file 1 — Data S1. Supporting Information. [file MRM-95-601-s001.pdf]

# Impact of Simultaneous Exposure to RF and Gradient Electromagnetic Fields on Implant MR Safety Labeling

Supporting Information

## ASTM Phantom

ASTM F2182 guidelines recommend the use of a 65 cm × 42 cm × 9 cm rectangular phantom based on a Polyacrylic acid (PAA) gelled-saline solution. The ISO 10974 standard does not elaborate on phantom geometry but suggests possible recipes for its preparation. Since the PAA gelled-saline solution is included in the ISO 10974 list, this was selected both for RF and GC simulations. The last row of Table S1 collects the electric and thermal properties of the simulated PAA-based phantom.

Table S1: Electric and thermal properties of the implant and testing phantom materials

| Material | Electrical Conductivity ($MS\,m^{-1}$) | Electric Relative Permittivity | Thermal Conductivity ($W\,m^{-1}\,K^{-1}$) | Specific Heat Capacity ($J\,kg^{-1}\,K^{-1}$) | Mass Density ($kg\,m^{-3}$) |
|---|---|---|---|---|---|
| Ti–6Al–4V | 0.595 | - | 6.6 | 580 | 4420 |
| CrCoMo | 1.160 | - | 14.00 | 450 | 8445 |
| Stainless Steel | 1.250 | - | 15.00 | 500 | 7900 |
| UHMWPE | 0 | 2.3 | 0.47 | 1900 | 940 |
| PAA gelled-saline | $0.47 \times 10^{-6}$ | 78 | 0.54 | 4152 | 998 |

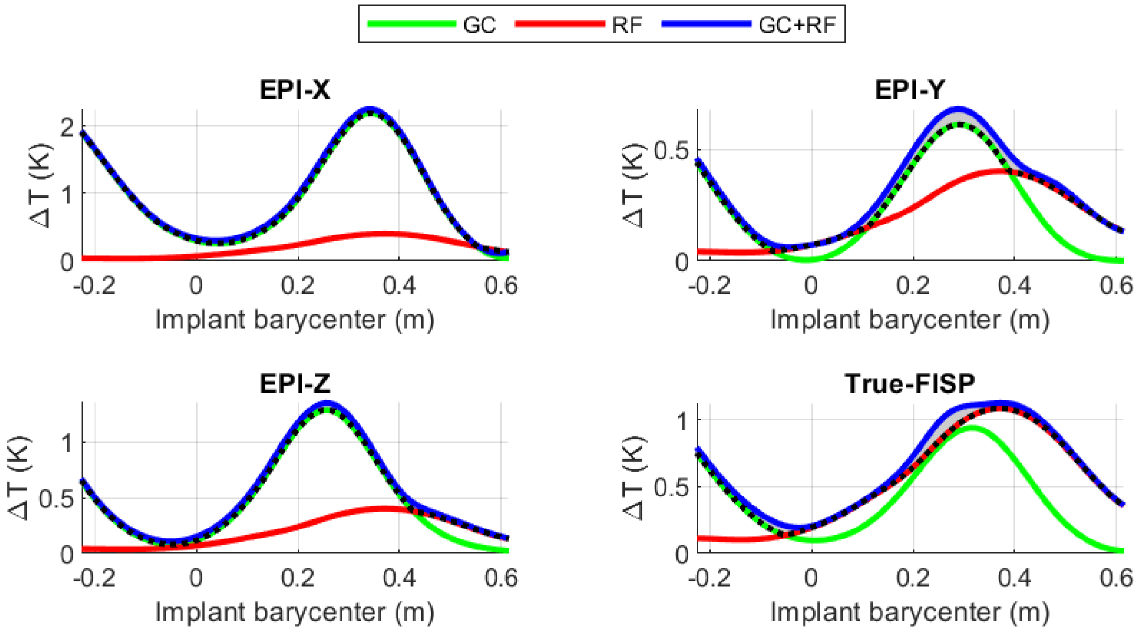


Figure S1: Peak temperature increase, in kelvin, on the shoulder implant exposed to different MR pulse sequences as a function of multiple axial landmarks. Results refer to 1.5 T exposure to the RF EM field alone (red line), GC EM field alone (green line) and simultaneous application of RF and GC EM fields (blue line). The gray area highlights the enhancement of the peak temperature increase due to the simultaneous exposure to RF and GC EM fields with respect to the maximum between the peak temperature increase due to RF or GC alone

Table S2: Relative enhancements of the maximum temperature increase due to the simultaneous application of GC and RF fields with the anatomical body models, with respect to the temperature increase due to GC or RF alone. For each implant and MR pulse sequence, RF and GC temperature increases were scaled to the same maximum value across the landmarks. The landmarks (i.e., the axial position of the implant barycenter with respect to the scanner isocenter) where the maximum temperature increases are reached by RF, GC and their combination are reported in the table.

| Implant | Sequence | Landmark of maximum temperature increase (m) | | | | | Relative enhancement (%) | |
|---|---|---|---|---|---|---|---|---|
| | | GC | RF | | Combined | | 1.5 T | 3 T |
| | | | 1.5 T | 3 T | 1.5 T | 3 T | | |
| AnklePlate | EPI-X | -0.47 | -0.82 | -0.82 | -0.47 | -0.47 | 0.52 | 0.95 |
| | EPI-Y | -0.55 | -0.82 | -0.82 | -0.55 | -0.55 | 0.12 | 1.35 |
| | EPI-Z | -0.47 | -0.82 | -0.82 | -0.47 | -0.47 | 1.05 | 1.56 |
| | FISP | -0.47 | -0.82 | -0.82 | -0.47 | -0.47 | 0.52 | 0.95 |
| CranialPlate | EPI-X | -1.49 | -1.34 | -1.34 | -1.49 | -1.49 | 1.53 | 4.36 |
| | EPI-Y | -1.49 | -1.34 | -1.34 | -1.49 | -1.49 | 1.72 | 4.11 |
| | EPI-Z | -1.42 | -1.34 | -1.34 | -1.42 | -1.42 | 0.85 | 2.85 |
| | FISP | -1.42 | -1.34 | -1.34 | -1.42 | -1.42 | 0.91 | 3.37 |
| Hip | EPI-X | -1.31 | -0.82 | -0.82 | -1.25 | -1.25 | 2.53 | 2.47 |
| | EPI-Y | -1.25 | -0.82 | -0.82 | -1.25 | -1.25 | 4.24 | 4.00 |
| | EPI-Z | -1.25 | -0.82 | -0.82 | -1.25 | -1.25 | 3.06 | 3.11 |
| | FISP | -1.31 | -0.82 | -0.82 | -1.31 | -1.31 | 1.85 | 1.70 |
| Knee | EPI-X | -0.41 | -0.83 | -0.55 | -0.41 | -0.41 | 5.70 | 10.19 |
| | EPI-Y | -0.83 | -0.83 | -0.55 | -0.83 | -0.83 | 8.17 | 4.49 |
| | EPI-Z | -0.83 | -0.83 | -0.55 | -0.83 | -0.83 | 4.20 | 4.97 |
| | FISP | -0.83 | -0.83 | -0.55 | -0.83 | -0.83 | 4.20 | 4.97 |
| SAIMD-U | EPI-X | -1.10 | -1.39 | -0.96 | -1.10 | -1.10 | 5.97 | 7.62 |
| | EPI-Y | -1.10 | -1.39 | -0.96 | -1.10 | -1.10 | 5.97 | 7.62 |
| | EPI-Z | -1.67 | -1.39 | -0.96 | -1.03 | -1.03 | 3.30 | 4.55 |
| | FISP | -1.10 | -1.39 | -0.96 | -1.10 | -1.10 | 5.96 | 7.61 |
| Shoulder | EPI-X | -1.06 | -0.99 | -1.06 | -1.06 | -1.06 | 7.58 | 7.20 |
| | EPI-Y | -1.13 | -0.99 | -1.06 | -1.13 | -1.13 | 8.49 | 9.57 |
| | EPI-Z | -1.13 | -0.99 | -1.06 | -1.13 | -1.13 | 7.42 | 8.47 |
| | FISP | -1.06 | -0.99 | -1.06 | -1.06 | -1.06 | 6.92 | 6.82 |

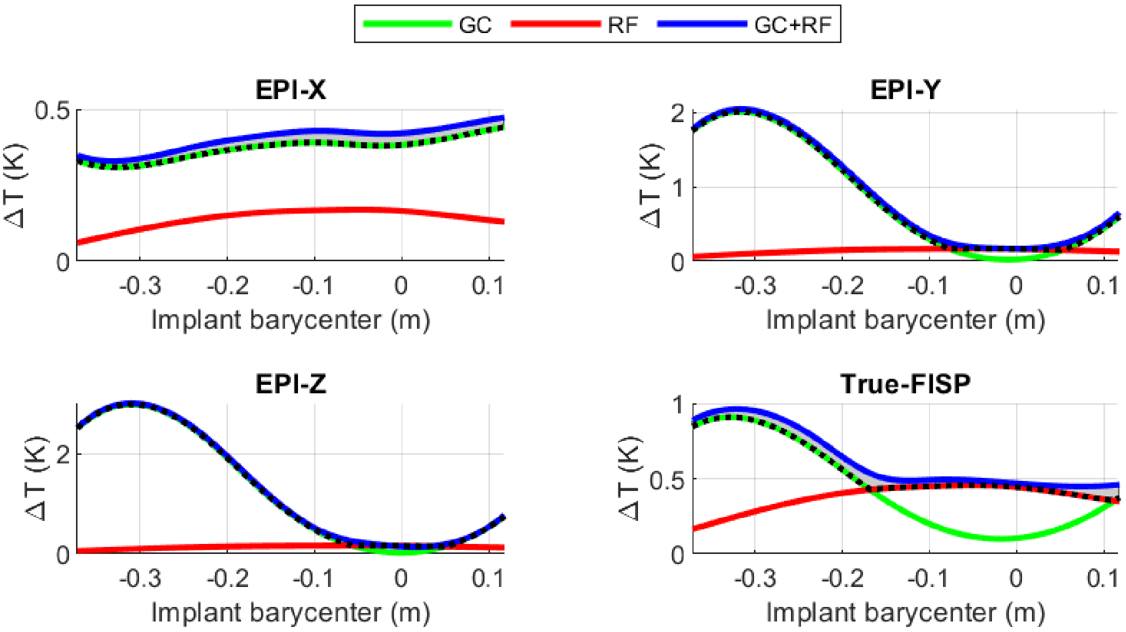


Figure S2: Peak temperature increase, in kelvin, on the knee implant exposed to different MR pulse sequences as a function of multiple axial landmarks. Results refer to 1.5 T exposure to the RF EM field alone (red line), GC EM field alone (green line) and simultaneous application of RF and GC EM fields (blue line). The gray area highlights the enhancement of the peak temperature increase due to the simultaneous exposure to RF and GC EM fields with respect to the maximum between the peak temperature increase due to RF or GC alone

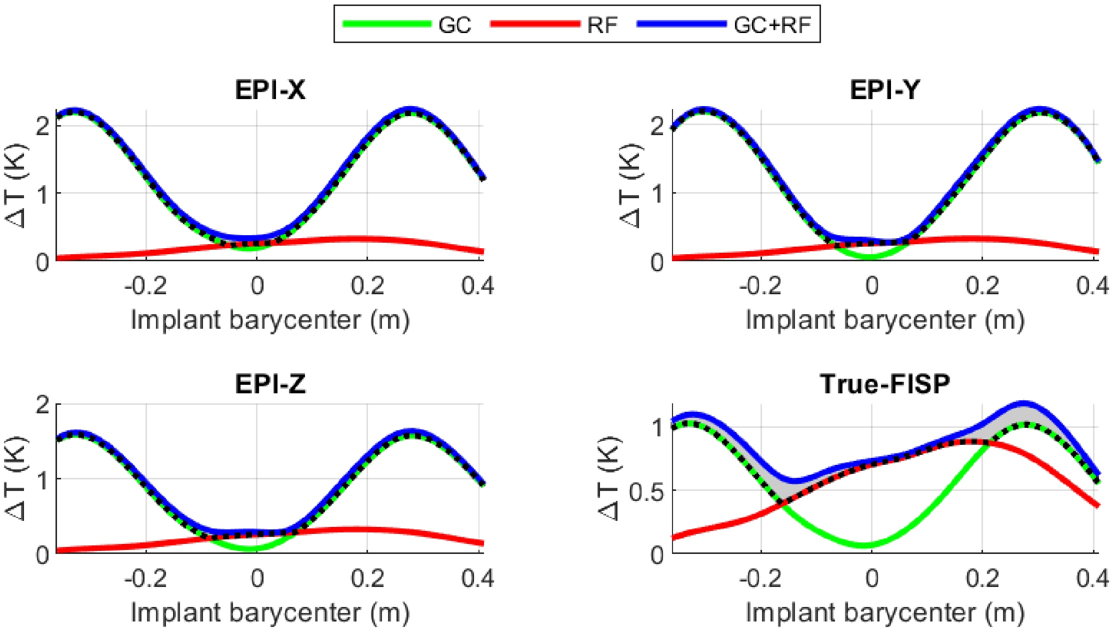


Figure S3: Peak temperature increase, in kelvin, on the hip implant exposed to different MR pulse sequences as a function of multiple axial landmarks. Results refer to 1.5 T exposure to the RF EM field alone (red line), GC EM field alone (green line) and simultaneous application of RF and GC EM fields (blue line). The gray area highlights the enhancement of the peak temperature increase due to the simultaneous exposure to RF and GC EM fields with respect to the maximum between the peak temperature increase due to RF or GC alone

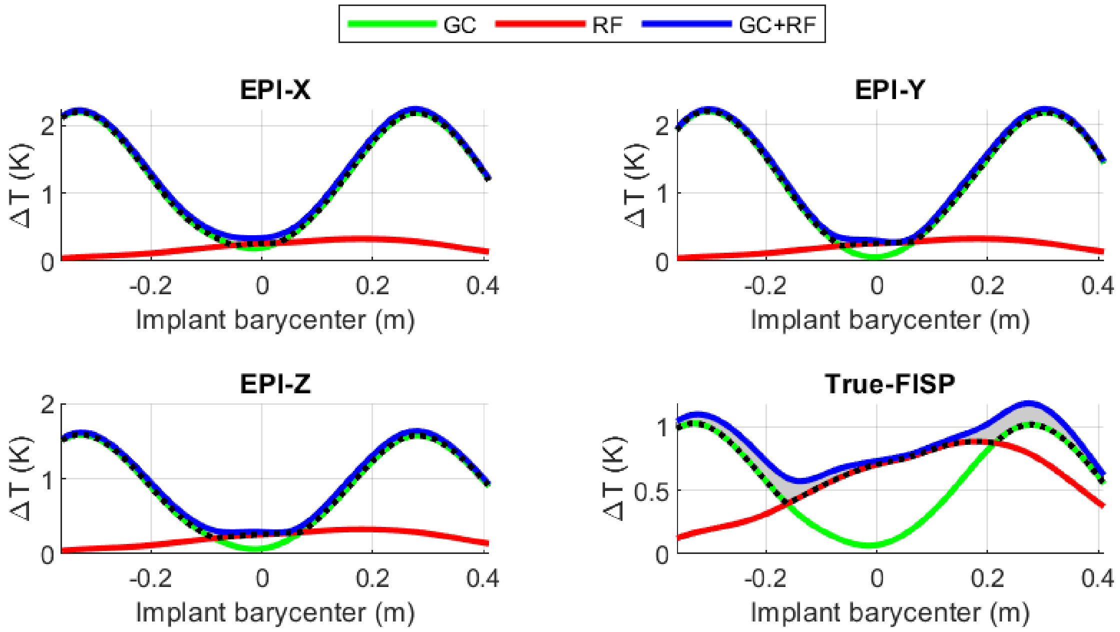


Figure S4: Peak temperature increase, in kelvin, on the ankle plate implant exposed to different MR pulse sequences as a function of multiple axial landmarks. Results refer to 1.5 T exposure to the RF EM field alone (red line), GC EM field alone (green line) and simultaneous application of RF and GC EM fields (blue line). The gray area highlights the enhancement of the peak temperature increase due to the simultaneous exposure to RF and GC EM fields with respect to the maximum between the peak temperature increase due to RF or GC alone

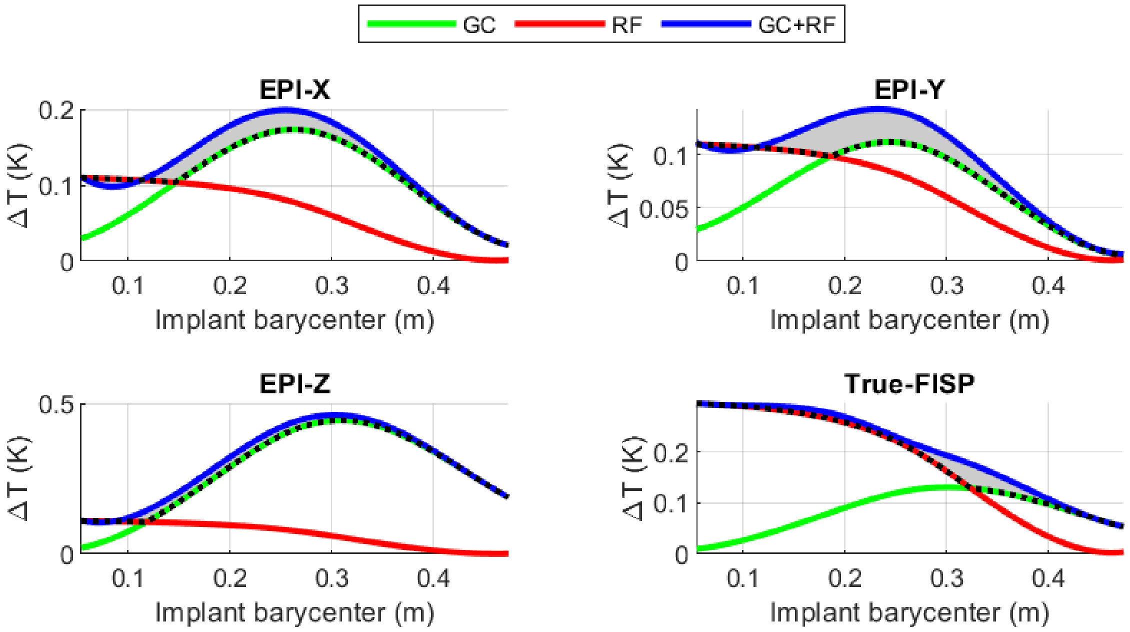


Figure S5: Peak temperature increase, in kelvin, on the cranial plate implant exposed to different MR pulse sequences as a function of multiple axial landmarks. Results refer to 1.5 T exposure to the RF EM field alone (red line), GC EM field alone (green line) and simultaneous application of RF and GC EM fields (blue line). The gray area highlights the enhancement of the peak temperature increase due to the simultaneous exposure to RF and GC EM fields with respect to the maximum between the peak temperature increase due to RF or GC alone

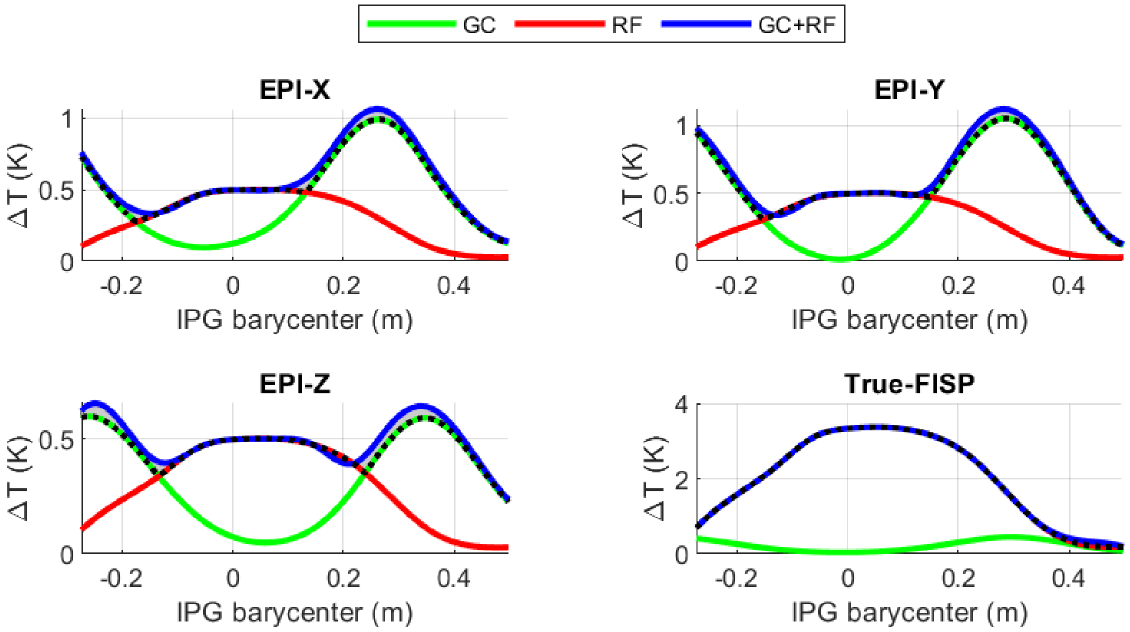


Figure S6: Peak temperature increase, in kelvin, on the SAIMD-U implant exposed to different MR pulse sequences as a function of multiple axial landmarks. Results refer to 1.5 T exposure to the RF EM field alone (red line), GC EM field alone (green line) and simultaneous application of RF and GC EM fields (blue line). The gray area highlights the enhancement of the peak temperature increase due to the simultaneous exposure to RF and GC EM fields with respect to the maximum between the peak temperature increase due to RF or GC alone

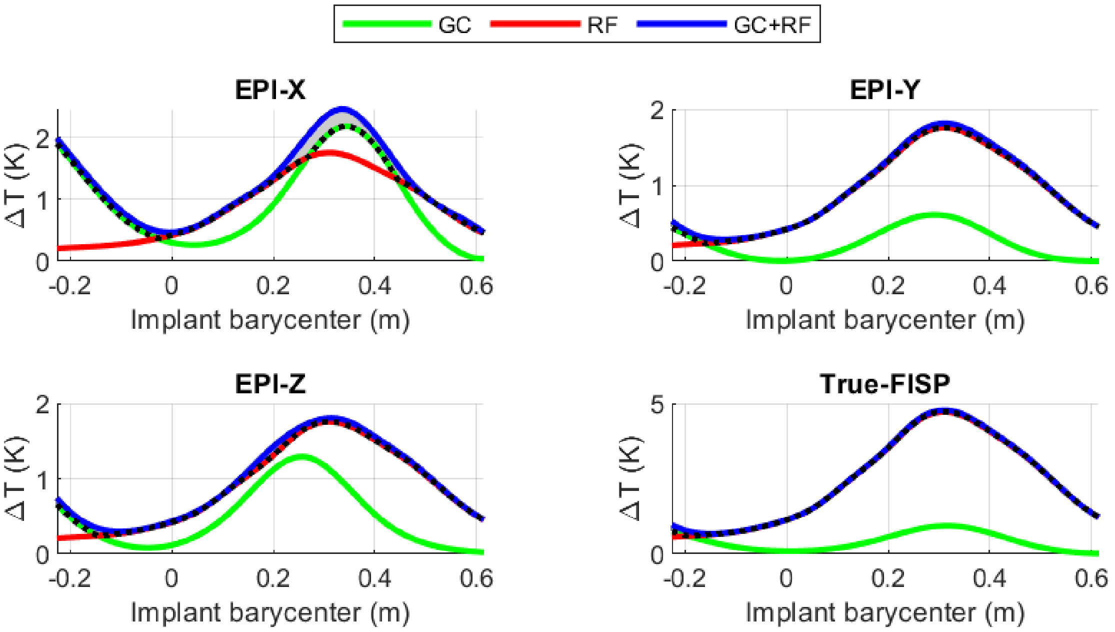


Figure S7: Peak temperature increase, in kelvin, on the shoulder implant exposed to different MR pulse sequences as a function of multiple axial landmarks. Results refer to 3 T exposure to the RF EM field alone (red line), GC EM field alone (green line) and simultaneous application of RF and GC EM fields (blue line). The gray area highlights the enhancement of the peak temperature increase due to the simultaneous exposure to RF and GC EM fields with respect to the maximum between the peak temperature increase due to RF or GC alone

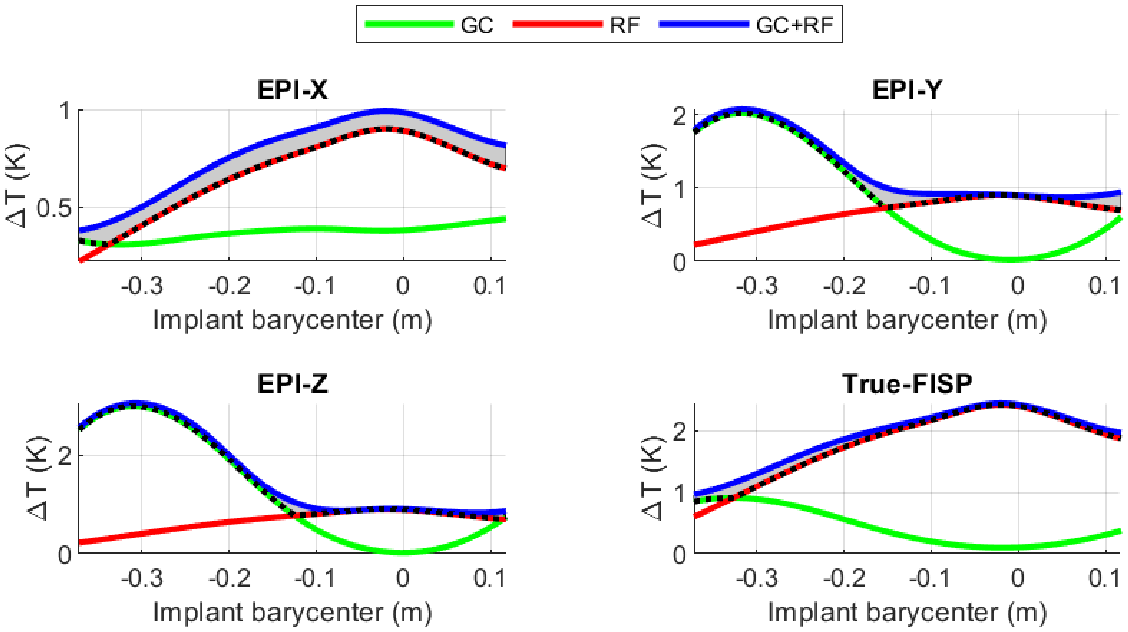


Figure S8: Peak temperature increase, in kelvin, on the knee implant exposed to different MR pulse sequences as a function of multiple axial landmarks. Results refer to 3 T exposure to the RF EM field alone (red line), GC EM field alone (green line) and simultaneous application of RF and GC EM fields (blue line). The gray area highlights the enhancement of the peak temperature increase due to the simultaneous exposure to RF and GC EM fields with respect to the maximum between the peak temperature increase due to RF or GC alone

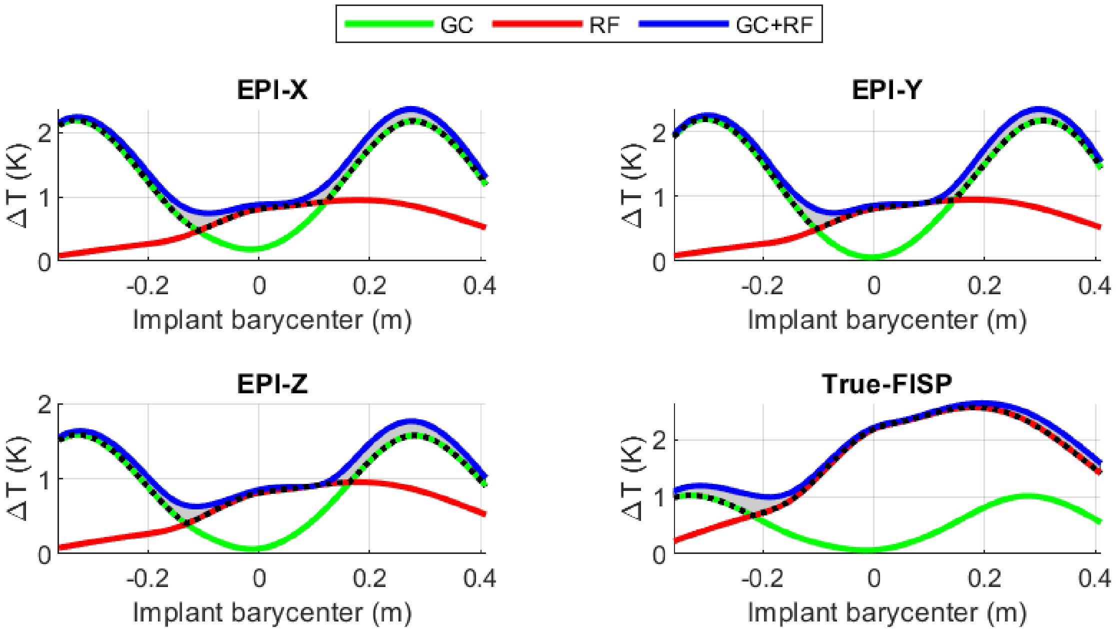


Figure S9: Peak temperature increase, in kelvin, on the hip implant exposed to different MR pulse sequences as a function of multiple axial landmarks. Results refer to 3 T exposure to the RF EM field alone (red line), GC EM field alone (green line) and simultaneous application of RF and GC EM fields (blue line). The gray area highlights the enhancement of the peak temperature increase due to the simultaneous exposure to RF and GC EM fields with respect to the maximum between the peak temperature increase due to RF or GC alone

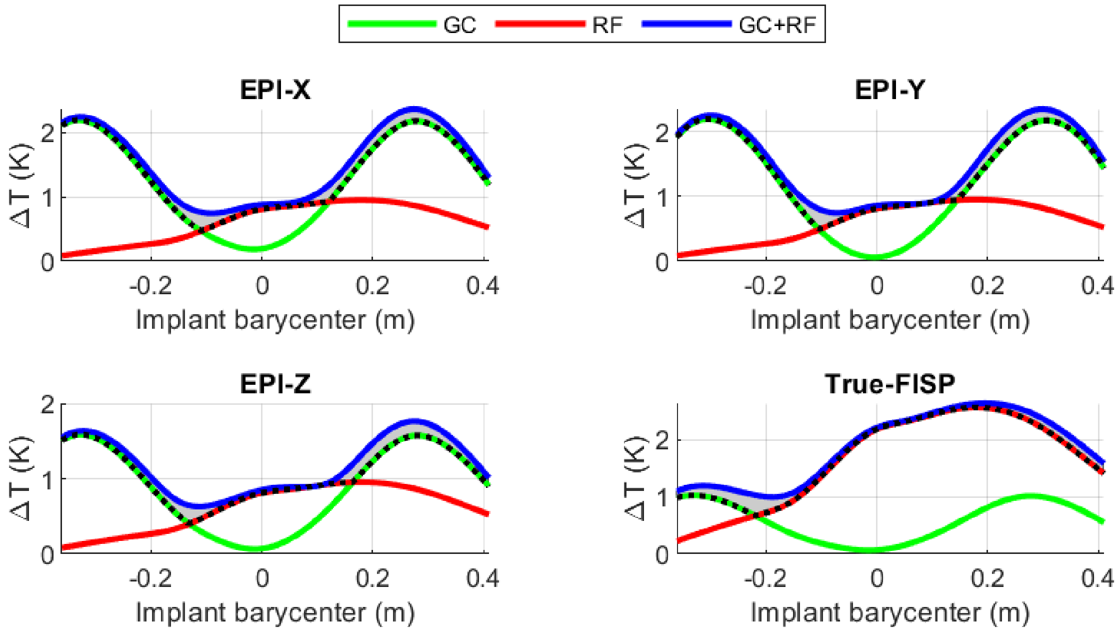


Figure S10: Peak temperature increase, in kelvin, on the ankle plate implant exposed to different MR pulse sequences as a function of multiple axial landmarks. Results refer to 3 T exposure to the RF EM field alone (red line), GC EM field alone (green line) and simultaneous application of RF and GC EM fields (blue line). The gray area highlights the enhancement of the peak temperature increase due to the simultaneous exposure to RF and GC EM fields with respect to the maximum between the peak temperature increase due to RF or GC alone

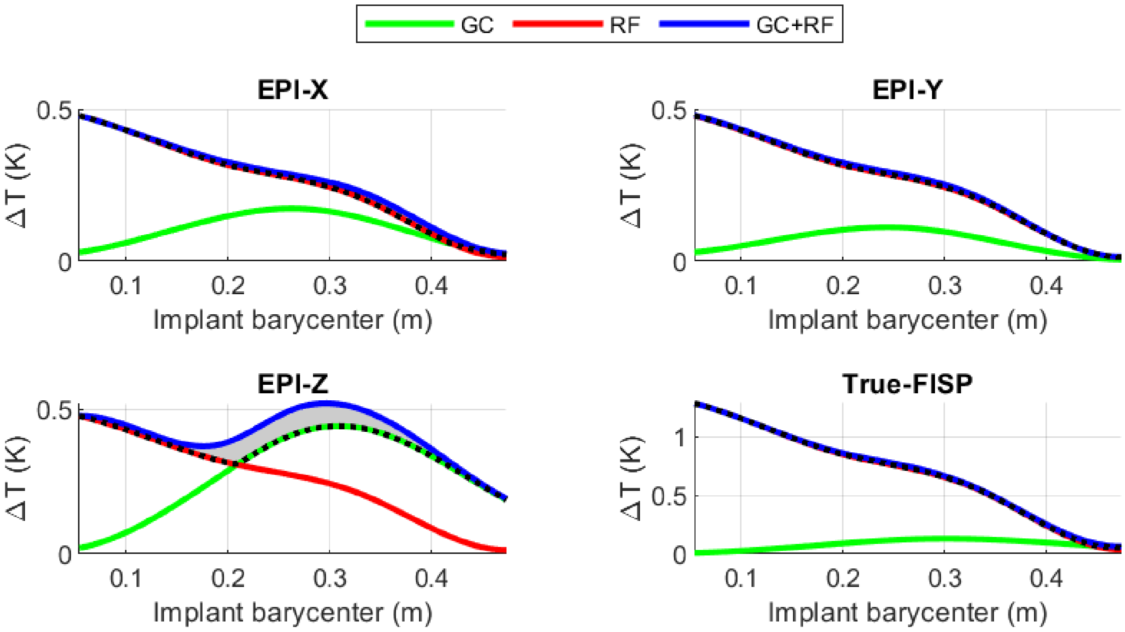


Figure S11: Peak temperature increase, in kelvin, on the cranial plate implant exposed to different MR pulse sequences as a function of multiple axial landmarks. Results refer to 3 T exposure to the RF EM field alone (red line), GC EM field alone (green line) and simultaneous application of RF and GC EM fields (blue line). The gray area highlights the enhancement of the peak temperature increase due to the simultaneous exposure to RF and GC EM fields with respect to the maximum between the peak temperature increase due to RF or GC alone

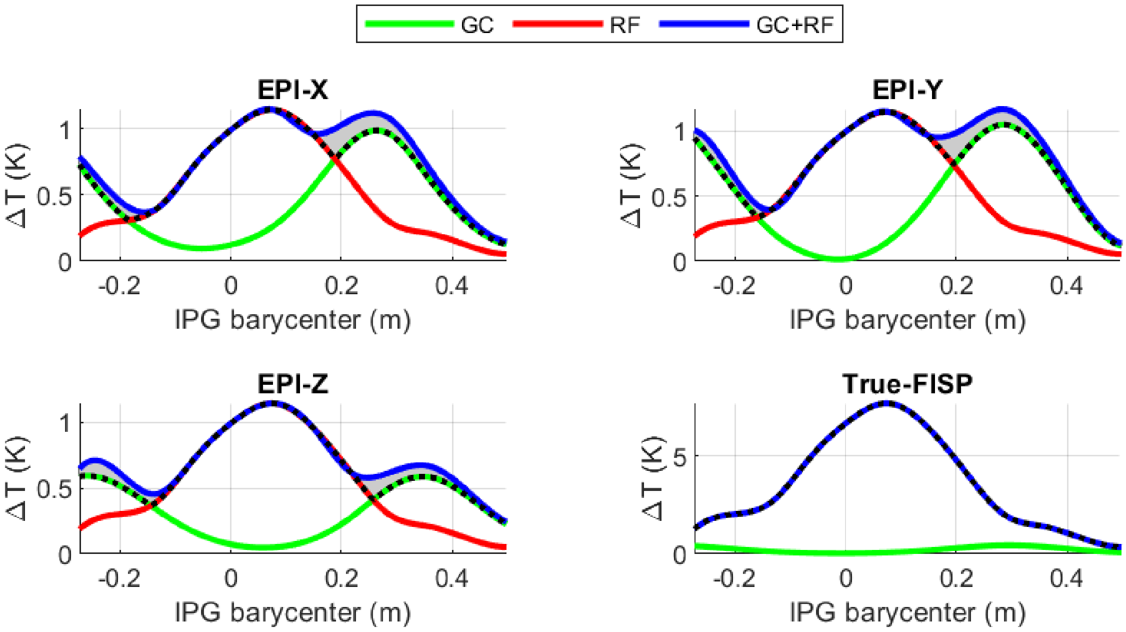


Figure S12: Peak temperature increase, in kelvin, on the SAIMD-U implant exposed to different MR pulse sequences as a function of multiple axial landmarks. Results refer to 3 T exposure to the RF EM field alone (red line), GC EM field alone (green line) and simultaneous application of RF and GC EM fields (blue line). The gray area highlights the enhancement of the peak temperature increase due to the simultaneous exposure to RF and GC EM fields with respect to the maximum between the peak temperature increase due to RF or GC alone
